# Supplementary material for: Microplastics in Cetaceans Stranded on the Portuguese Coast
Source: Animals (Basel). 2023 Oct 19;13(20):3263. doi: 10.3390/ani13203263 (PMC10603649; doi:10.3390/ani13203263)
Supplement: Supplementary file 1 [file animals-13-03263-s001.zip › animals-2641380-supplementary.pdf]

SUPPLEMENTARY INFORMATION

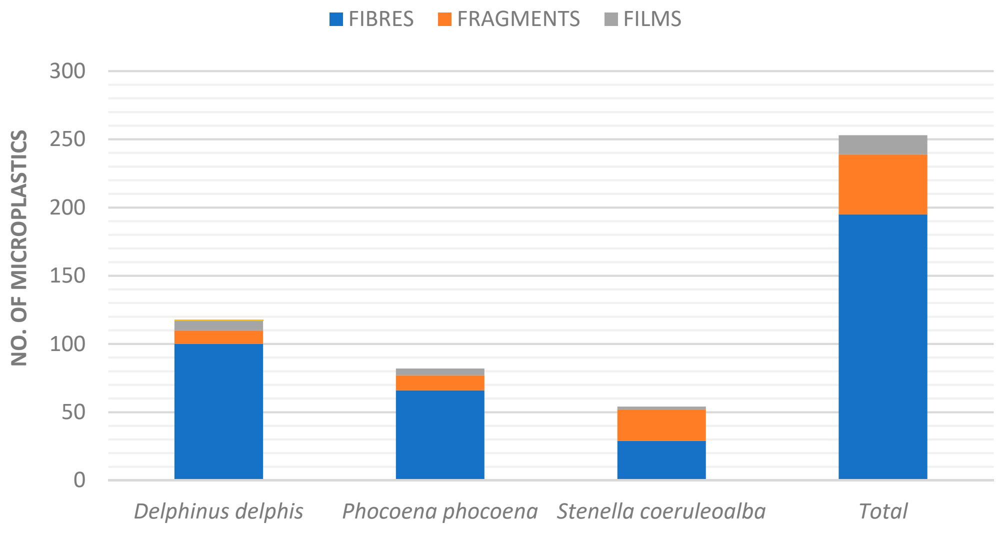

Figure S1. Number of microplastics of each category type for each of the analysed species and for all analysed individuals

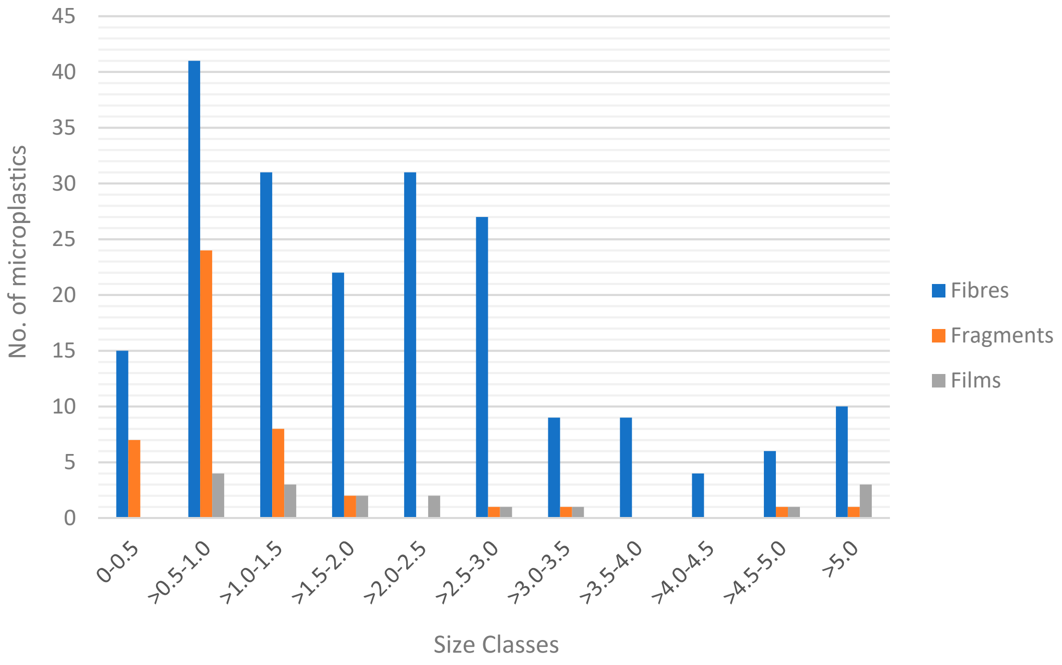

Figure S2. Number of plastic particles per size ranges (mm) in the analysed cetaceans. Note: mesoplastics (>5mm) were also included in the category >5.0 mm.

Microplastics in cetaceans stranded in the Portuguese coast: Supplementary Information

Table S1. Statistics of the Mann–Whitney U tests and Kruskall-Wallis tests used to assess the influence of explanatory variables on the number of microplastics (no. of microplastics) and number of fibres (no. of fibres) in all analysed species.

Explanatory variable	Test	Response variable	Results
Sex (Male/ Female)	Mann-Whitney U test	no. of microplastics	W = 209; p-value = 0.4118
		no. of fibres	W = 179, p-value = 0.9766
Maturity (Immature/ Mature)	Mann-Whitney U test	no. of microplastics	W = 106, p-value = 0.2553
		no. of fibres	W = 97.5, p-value = 0.447
Parasites (Presence/ Absence)	Mann-Whitney U test	no. of microplastics	W = 106.5, p-value = 0.9685
		no. of fibres	W = 113.5, p-value = 0.8432
Cause of death (Bycatch vs. Trauma/Disease)	Mann-Whitney U test	no. of microplastics	W = 132.5, p-value = 0.9434
		no. of fibres	W = 116, p-value = 0.631
Sampling years (2017-2019)	Kruskall-Wallis	no. of microplastics	χ^2 = 0.64252; df = 2; p-value = 0.7252
		no. of fibres	χ^2 = 0.32723; df = 2; p-value = 0.8491
Age classes (Calves, juveniles and adults)	Kruskall-Wallis	no. of microplastics	χ^2 = 1.1114; df = 2; p-value = 0.5737
		no. of fibres	χ^2 = 0.58247; df = 2; p-value = 0.7473
Body condition (Good, moderate and thin)	Kruskall-Wallis	no. of microplastics	χ^2 = 0.37064; df = 2; p-value = 0.8308
		no. of fibres	χ^2 = 1.0458; df = 2; p-value = 0.5928

Table S2. Statistics of the Mann–Whitney U tests and Kruskall-Wallis tests used to assess the influence of explanatory variables on the number of microplastics (no. of microplastics) and number of fibres (no. of fibres) in analysed common dolphins.

Explanatory variable	Test	Response variable	Statistics
Sex (Male/ Female)	Mann-Whitney U test	no. of microplastics	W = 87, p-value = 0.3989
		no. of fibres	W = 66.5, p-value = 0.7705
Maturity (Immature/ Mature)	Mann-Whitney U test	no. of microplastics	W = 40.5, p-value = 0.6415
		no. of fibres	W = 41.5, p-value = 0.6928
Parasites (Presence/ Absence)	Mann-Whitney U test	no. of microplastics	W = 63, p-value = 0.6621
		no. of fibres	W = 61.5, p-value = 0.7355
Cause of death (Bycatch vs. Trauma/Disease)	Mann-Whitney U test	no. of microplastics	W = 18.5, p-value = 0.1012
		no. of fibres	W = 23.5, p-value = 0.2105
Sampling years (2017-2019)	Kruskall-Wallis	no. of microplastics	χ^2 = 0.28831; df = 2; p-value = 0.8658
		no. of fibres	χ^2 = 0.60514; df = 2; p-value = 0.7389
Age classes (Calves, juveniles and adults)	Kruskall-Wallis	no. of microplastics	χ^2 = 1.0655; df = 2; p-value = 0.587
		no. of fibres	χ^2 = 2.1364; df = 2; p-value = 0.3436
Body condition (Good, moderate and thin)	Kruskall-Wallis	no. of microplastics	χ^2 = 0.95321; df = 2; p-value = 0.6209
		no. of fibres	χ^2 = 0.21333; df = 2; p-value = 0.8988
